# The Main Thing is to be Alive—Exploring Patients’ Experiences With Weight Gain After Liver Transplantation: A Qualitative Study

**DOI:** 10.3389/ti.2022.10256

**Published:** 2022-04-14

**Authors:** Sonja Beckmann, Patrizia Künzler-Heule, Kajetan Kabut, Oliver Mauthner

**Affiliations:** ^1^ Nursing Science, University of Basel, Basel, Switzerland; ^2^ Center of Clinical Nursing Science, University Hospital Zurich, Zurich, Switzerland; ^3^ Department of Gastroenterology/Hepatology, Cantonal Hospital St. Gallen, St. Gallen, Switzerland; ^4^ Department of Nursing, Cantonal Hospital St. Gallen, St. Gallen, Switzerland; ^5^ Zentrum für NeuroRehabilitation, Beatmungs- und Intensivmedizin, BDH-Klinik Elzach, Elzach, Germany; ^6^ University Department of Geriatric Medicine Felix Platter, Basel, Switzerland

**Keywords:** education, obesity, self-management, behavior change, communication, thematic analysis

## Abstract

Weight gain after liver transplantation (LTx) contributes to new-onset obesity. We explored patients’ experiences with gaining weight after LTx. Individual interviews were guided by open-ended questions. We analyzed transcripts with the reflexive thematic analysis approach by Braun and Clarke. The 12 participants gained 11.5 kg weight (median) over a median of 23 months after LTx. The constitutive theme “The main thing is to be alive” was a recurrent insight, captured in three facets: “The arduous path back to living” was the emotional expression of the ups and downs during a life-threatening illness to finally being grateful for the new life. “A pleasurable new phase of life” was the legitimation, reflecting the appreciation of gaining weight and returning to a healthy appearance. “I am allowed to look like this now” was the consoling facet after a time of burden due to the increased weight and frustration of being unsuccessful in losing weight. Finally, the awareness of being a LTx survivor outplayed the burden of the excess weight. Early interventions are crucial because the comforting insight “I am allowed to look like this now” may hinder further engagement in weight loss activities. Our recommendations on education and self-management support may guide clinical practice.

## Graphical Abstract

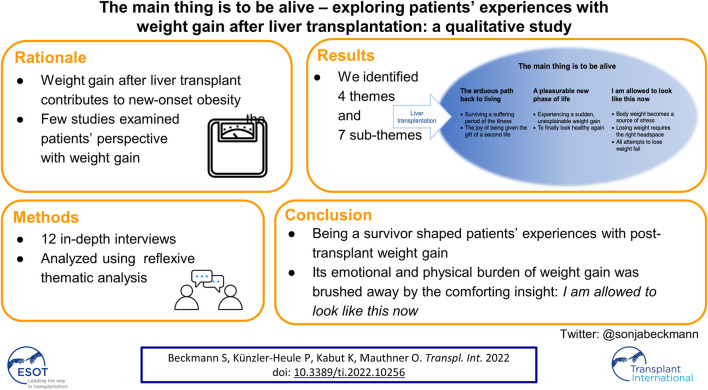

## Introduction

Weight gain after liver transplantation (LTx) has been a research focus for over 30 years (1). Studies report a mean weight gain between 2 and 9 kg within the first year post-LTx (2-4). At 2- and 3-year following transplantation, continuous weight gain contributes to new-onset obesity in 22%–38% of patients (2–4). Post-LTx weight gain and new-onset obesity contribute to increased long-term mortality (5) and comorbidities such as metabolic syndrome (6), non-alcoholic fatty liver disease (7) and cardiovascular events (8). These significant weight gains and risks prompt the question of how patients cope with this situation. Unfortunately, few studies examined the patients’ perspective (1, 9, 10).

The etiology of weight gain is complex and depends on a multitude of individual and interconnected factors (11). Previous studies suggested that post-LTx weight gain was related to higher weight or body mass index (BMI) pre-LTx, being a former smoker, older age at LTx (2), alcoholic liver disease as reason for LTx (2, 8) and genetic factors (12). Interestingly, one study found no association between energy intake or daily physical activity and overweight or obesity after LTx (13). This contrasts two quantitative studies that added unstructured qualitative questions to their data collection, asking patients about the causes of their post-LTx weight gain (1, 9). Constant hunger with increased food intake and reduced daily physical activity were among the most common answers. Although those results should be interpreted cautiously due to methodological weaknesses, they are supported by a study exploring the perceptions of 20 patients on weight gain after LTx (10). That analysis revealed several reasons for weight gain including behavioral factors (e.g., diet, improved health/appetite, sedentary behavior) and other factors such as medication, weight regain, older age and addiction. To prevent weight gain, patients emphasized the need for supportive group programs, consultation with a dietician and advice for future recipients. Delivering supportive advice only is, however, not necessarily effective. A study examined the impact of lifestyle advice among overweight or obese patients after LTx (14). Despite being advised to lose weight, 62% gained weight (median 4.0 kg) during the study period. The authors concluded that simply reiterating the importance of following guidelines was ineffective for LTx recipients. Indeed, evidence suggests that effective weight loss interventions should understand predictors of behavior such as motivation, opportunity and capability (15) and also account for a person’s readiness to change this behavior. A well-known framework to explain the stages of changing a behavior is the transtheoretical model (TTM) (16, 17). Each stage of the TTM requires a distinct intervention approach and it has been frequently used in dietary or physical activity weight loss interventions (18).

Given the research on the evolution and impact of post-LTx weight gain, there is a surprising lack of high-quality evidence on the content, delivery, timing and efficacy of weight management interventions (19, 20). This study therefore explored how patients experienced weight gain after LTx. *In-depth* knowledge of the lived experience, beliefs and motivations provides information to improve patient care and for developing interventions after LTx.

## Methods

### Sample

Participants were recruited at the University Hospital Zurich with following inclusion criteria: ≥18 years, german speaking, ≥5 kg weight gain between LTx until recruitment, and ≥12 months since LTx. Participants were purposefully selected on gender, age and time since LTx to ensure a heterogeneous study group allowing diverse perspectives to be explored. The Cantonal Ethics Committee Zurich approved the study (BASEC 2017-01429).

The first author (SB) worked in the LTx nurse counseling service and identified possible participants from the hospital’s electronic patient charts. Measuring weight is a routine procedure in the hospital’s follow-up visits. Patients who gained at least 5 kg from LTx until the latest follow-up visit were approached *via* telephone or face-to-face to provide oral and written study information. The contact information of interested persons was transferred to another author (KK), who conducted the interviews and was not involved in caring for the patients.

Fourteen people were asked to participate; two people declined. After providing a written informed consent, participants were interviewed in a place convenient for them, either in hospital or at home. The interviewer followed a guideline with open-ended questions to encourage the participants to share their experiences. The interview started with the question: “People report that their lives changed after LTx. Could you please tell me how your daily life and routine have changed since the LTx?” The subsequent questions explored more specific experiences with gaining weight, eating or activity, such as “What effects did the weight gain have on your everyday life?”

### Data Collection and Analysis

Individual interviews were conducted between September 2017 and June 2018, lasting between 29 and 84 min (mean: 47 min). They were conducted in German, digitally recorded, transcribed and pseudonymized. Field notes were made during and after the interview. The research group consisted of a junior researcher and three senior researchers with expertise in qualitative research and clinical care of transplant patients. SB and KK listened to all interviews and read all transcripts. The other members listened to and read selected interviews or text passages for trustworthiness. Codes and themes were discussed in the group throughout the analysis, and final results were discussed with two interviewees for feedback. Sociodemographic and clinical data were self-reported by the participants before the interviews.

Data analysis followed the six phases of Braun and Clarke’s reflexive thematic analysis (21, 22), which identifies, analyses and reports patterns (themes) of shared meaning: 1) Familiarizing with the data by transcribing the interviews, re-reading and taking notes of thoughts and interesting characteristics. 2) Generating codes by identifying meaningful text passages. 3) Constructing themes by grouping and naming the coded data that were related to each other. 4) Revising themes to clarify the scope and avoid confusion or overlap. Checking the fit of the themes against each other and the dataset. 5) Final definition and naming of themes to ensure clarity and comprehensiveness. 6) Writing the article as a final check if the themes made sense and would answer the research question by telling a coherent story. Data were managed using the computer software MAXQDA, Release 20.0.8 (Verbi GmbH, Berlin, Germany). The reporting followed the Consolidated Criteria for Reporting Qualitative Health Research (COREQ) Checklist (Supplementary Material S1) (23).

## Results

**FIGURE 1:**
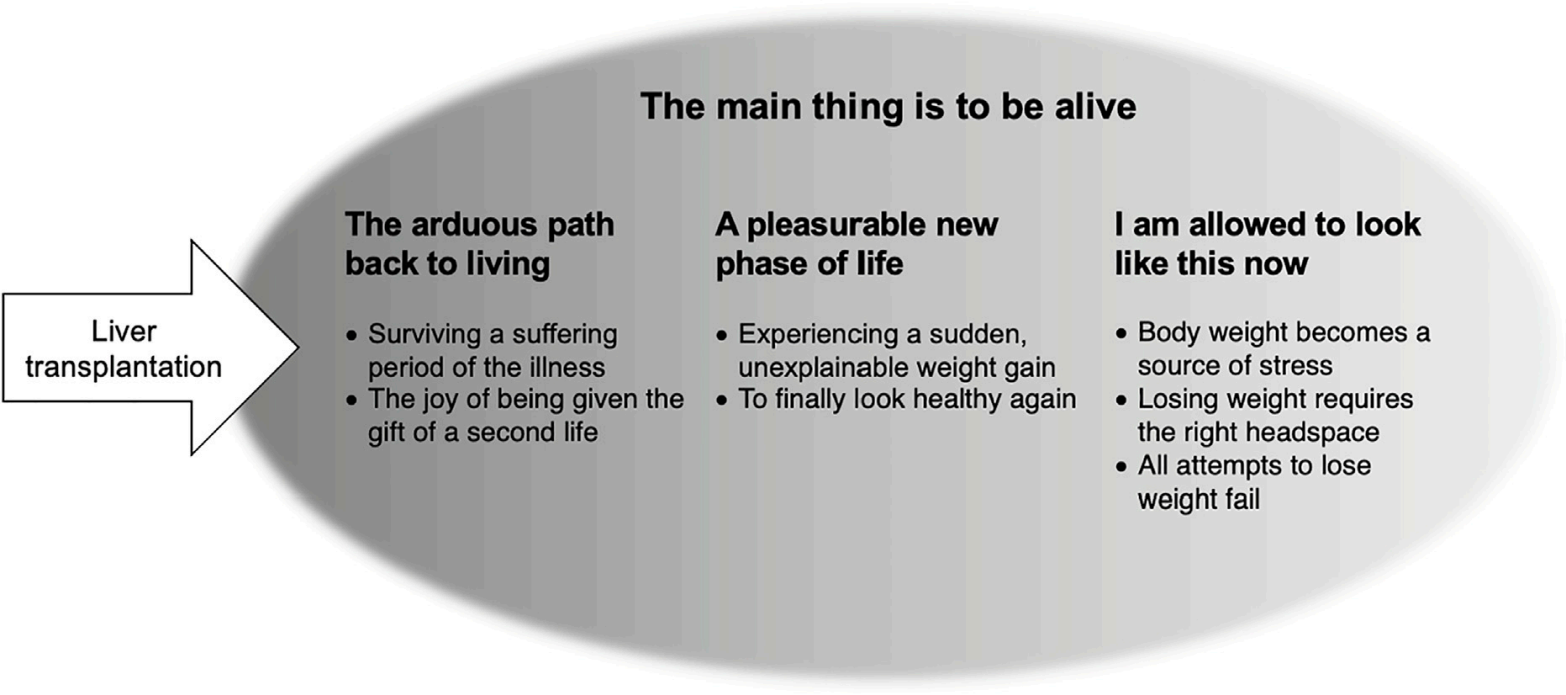
Overview over the four themes and the seven subthemes.

Twelve participants shared their experiences of post-LTx gaining weight. Median time since LTx was 23 months (range 17–58 months), individual weight gain ranged between 5 kg and 24 kg (median 11.5 kg). The participants were equally distributed in three groups: normal weight, overweight or obese at time of interview. Characteristics are shown in Table 1. We identified four themes and seven sub-themes, depicted in Figure 1. Representative quotes are in the text and Table 2.

**TABLE 1:** Sociodemographic and clinical data.

Pseudonym	Gender, age at interview	Months after LTx	Reason for LTx	Weight category (BMI) in 1st follow-up after LTx	Weight gain from 1st follow-up after LTx until interview (kg)	Weight category (BMI) at time of interview
Joe	Male, 70	18	Hepatocellular carcinoma	Obesity (34.5 kg/m^2^)	8	Obesity (37.1 kg/m^2^)
Marlene	Female, 55	31	Alcoholic liver cirrhosis	Normal weight (24.5 kg/m^2^)	24	Obesity (32.9 kg/m^2^)
Norbert	Male, 72	17	Primary sclerosing cholangitis	Normal weight (20.4 kg/m^2^)	11	Normal weight (23.9 kg/m^2^)
Kitty	Female, 23	22	Re–LTx, acute on chronic liver failure	Normal weight (19 kg/m^2^)	9	Normal weight (22.0 kg/m^2^)
Elisabeth	Female, 55	29	Liver cirrhosis after autoimmune hepatitis	Normal weight (18.8 kg/m^2^)	8	Normal weight (22.2 kg/m^2^)
Lara	Female, 58	71	Primary biliary cirrhosis	Normal weight (21.6 kg/m^2^)	18	Overweight (27.7 kg/m^2^)
Margret	Female, 67	51	Hepatocellular carcinoma	Obesity (33.3 kg/m^2^)	11	Obesity (37.3 kg/m^2^)
Rudi	Male, 62	58	Alcoholic liver cirrhosis	Normal weight (20.5 kg/m^2^)	20	Overweight (27.6 kg/m^2^)
Angelo	Male, 68	21	Alcoholic liver cirrhosis	Normal weight (20.1 kg/m^2^)	12	Normal weight (24.4 kg/m^2^)
Valérie	Female, 45	24	Autoimmune hepatitis	Normal weight (22.7 kg/m^2^)	24	Obesity (30.7 kg/m^2^)
Katja	Female, 43	16	Acute liver failure	Overweight (21.6 kg/m^2^)	14	Overweight (26.6 kg/m^2^)
Hans	Male, 64	22	Hepatocellular carcinoma	Overweight (24.8 kg/m^2^)	8	Overweight (27.2 kg/m^2^)

LTx, liver transplant; BMI, body mass index.

**TABLE 2:** Additional representative quotes within the themes.

Theme	Representative quotes
**The arduous path back to living**	«I was so exhausted after showering that I could barely towel myself dry. It’s hard to imagine, but that’s how it was.» (Rudi)
Surviving a suffering period of the illness	«I was traumatized as well. […] These 4 weeks in a coma left me very weak physically. […] And then came the period after the transplantation when I was in an induced coma […] When you are informed of that afterwards when you’re conscious again, that really is something that takes some time to work through. It took me a long time to regain my strength, it went on for 2 years for sure.» (Lara)
The joy of being given the gift of a second life	«Even now there are limits to what I can do because I just don’t have the stamina or the strength.» (Katja)
	«I have to plan ahead if I want to go somewhere: What will I be doing the following day? If I do whatever it is, will I be too tired to manage to go to work the next day?» (Marlene)
**A pleasurable new phase of life**	«After the transplantation things slowly got better, but it took a while for hunger pangs to return to normal. But after that I was really ravenously hungry […] I had the feeling that I had to make up for everything I had missed in the previous months.» (Marlene)
Experiencing a sudden, unexplainable weight gain	«Eating should be a pleasure» (Hans)
To finally look healthy again	«Before my transplantation, I had to take my temperature every day, weigh myself, and measure my blood pressure. I’m still a bit traumatized. I’m done with scales.» (Lara)
	«Ate too much? Lounged around too much? No idea, I don’t know. […] All of a sudden it was more. There’s no accounting for it.» (Margret)
	«I haven’t felt this good in ages.» (Angelo)
	«They (family) accept me the way I am and are glad that I’m healthy. That’s what’s most important to them.» (Marlene)
**I am allowed to look like this now**	«I get too little exercise, it’s true. Sometimes because I also have a nap in the afternoon, even in nice weather. I’m not making excuses, that’s just how it is.» (Joe)
Body weight becomes a source of stress	«I feel better and everything is back to working the way it should.» (Katja)
Losing weight requires the right headspace	«I’m at a standstill with my weight, basically 10 kg overweight. It’s apparent to me that it puts a great strain on me. And it enrages me because there’s nothing I can do about it. […] But I can’t change anything about it. And that’s what makes me kind of sad and crazy at the same time.» (Lara)
All attempts to lose weight fail	«Whenever I was at the hospital (for a follow-up), they said: Oh, you’ve put on weight, that’s good. And then suddenly it was: Stop, no more! You really have to watch your weight now! Oh, okay. Now what am I supposed to do: eat or not eat? Yeah, that was pretty stupid.» (Valérie)
	«(It doesn’t bother me that I) am a bit overweight. That’s just how it is. No, I have to look like this. […] They (the family) see me as an individual and not as an overweight person. They say: You’re still here. Never mind.» (Valérie)

### The Main Thing is to be Alive

This theme was identified as constitutive as it represented the most definitive and recurring insight into weight gain post-LTx: A recurring insight because it was captured in each of three other themes. Shortly after the LTx, it exemplified the manifold emotions generated by the theme *The arduous path back to living*. This was followed by a period in which those affected felt justified in indulging themselves, the theme *A pleasurable new phase of life* began. Then, after experiencing burden due to the increased weight and when all attempts to lose weight were unsuccessful, came the consoling theme *I am allowed to look like this now*. The three themes followed one another chronologically, although not all participants contributed to the final theme. The transition between themes was individual, depending on the recovery process and post-LTx weight attained.

### The arduous Path Back to Living

#### Surviving a Suffering Period of the Illness

The participants dramatically described surviving a severe liver disease, the life-saving LTx and subsequent recovery. Some experienced concomitant complications such as multiple organ failure or organ rejection. Nearly all described the regeneration process as arduous, with daily life marked by physical limitations, difficulty concentrating and extreme, ongoing fatigue. The participants’ physical ailments were compounded by emotional distress. They felt anxiety and uncertainty regarding transplantation success and were confronted with their own mortality. This long, energy-sapping phase left its mark psychologically. In Lara’s case it led to psychological trauma, for which she was still in treatment.

While the participants experienced emotional and physical improvement over time and reported a «slow return to living» (Lara), this did not always succeed to the extent expected or wished for. At the time of the interviews, almost no participants had regained the same level of stamina or strength they had had previously. In order to not overtax themselves, individuals had to be mindful of their own energy reserves and to use them carefully to best deal with the «arduous daily life» (Valérie).

#### The Joy of Being Given the Gift of a Second Life

Although, for some, ongoing physical problems and experiences continued to be emotionally stressful, the participants all looked back positively on this period. Any enduring limitations were accepted with equanimity, including several participants concluding that their bodies simply were «no longer what they had once been» (Lara). Generally, a feeling of joy at still being alive predominated, with many participants describing the great happiness they had experienced. Margret’s transplantation was due to hepatic cancer. She had been symptom free and «had been rather lucky» that her cancer was discovered in time. Angelo felt it was an «immense luck» that he had received a donor organ. The euphoria and gratitude for this «gift of a second life» (Elisabeth) accompanied the participants afterwards.

### A Pleasurable new Phase of Life

The participants conveyed a foundational positivity as they spoke of their joie de vivre and their desire to make the most of this phase of life. The enjoyment also manifested itself at mealtimes. After the lack of appetite and the deprivations experienced earlier, many participants reported feeling intense hunger. They had the feeling that food was a means of making up for something. Going grocery shopping, the preparation and enjoyment of meals once again became an integral part of daily life. Nutrition was often mentioned in connection with the belief that, having overcome the illness, they were justified in indulging themselves and were entitled to enjoy something pleasurable. Angelo, for example, consumed up to 1.5 L of sugar-sweetened soft drinks daily and took the view that «I didn’t undergo an operation just so that I could restrict myself to water.»

#### Experiencing a Sudden, Unexplainable Weight Gain

Along with pleasurable indulgence came weight gain. Only Norbert, Kitty und Elisabeth were all consciously aware of the process of weight gain. Upon reaching their pre-LTx weight, they reminded themselves «to having to exercise self-discipline» (Norbert) to avoid further weight gain. In contrast, most participants first became aware of having gained weight when their clothes grew tighter. Almost no-one had weighed themselves regularly, regarding it as unnecessarily stressful or as reminding them too much of when they were ill. In the absence of regular weighing, they were taken aback once they stood on scales; it was just so «sudden» (Marlene) and happened «at lightning speed» (Rudi).

In retrospect, the participants self-identified potential reasons for their weight gain: menopause, hormones, age, giving up smoking, and some spoke of a voracious appetite due to cortisone therapy. In terms of eating and exercise, one opinion was nearly universally held: «I don’t think it’s from eating but rather from a lack of exercise» (Valérie). Participants did not perceive any change in their eating habits over time. Only one person thought he ate larger portions than before, while all others made the point that «really, I just eat normally» (Rudi). However, almost everybody estimated their own level of exercise as being too low. Only a few took part in sporting activities and everyday activity was restricted, in particular, because pain or fatigue kept them at home, they had become unemployed or socialized less often. Looking back, no-one had a clear explanation for what exactly had caused the weight gain.

#### To Finally Look Healthy Again

Overall, weight gain was welcome and positively assessed by all participants because everybody had lost weight pre- and post-LTx. Some had undergone extreme weight loss, greatly impacting their physical appearance, at times amounting to little more than «skin and bones» (Katja), which was «not a pretty sight» (Lara). To be thin and ill-looking was stressful, and all participants were glad when this phase had ended. Many noted that their muscles were regaining strength, they had more energy and a better quality of life. They emphasized that they felt better overall, regardless of whether they had reached their normal weight or had become overweight or obese. The participants’ perceptions of their reinvigorated and well-functioning bodies reinforced this positive attitude. A severe illness had been overcome and weight gain was a visible and tangible representation of a return to health. They were back in the fullness of life. Family and friends reinforced this positive attitude towards weight gain. In this instance also, it did not matter whether the weight gain led to overweight or obesity. The most important thing was that the individual affected had survived the disease and was healthy once again.

### I am Allowed to Look Like This Now

#### Body Weight Becomes a Source of Stress

For those who did not halt their weight gain deliberately and in a timely manner, what had been an emotionally reassuring and positive experience turned into a stressful one. Those affected felt uncomfortable with their weight, experienced it as unpleasant or were «ashamed» (Lara). The weight category they fell into made no difference. Angelo was normal weight and dissatisfied after gaining five kilos in 4 months. Marlene was particularly adversely affected by her new-onset obesity: «It bothers me a lot the way it looks. […] Sometimes I have bad days where everything tends to go wrong. […] You get home in the evening totally wiped out, and then I look at myself in the mirror and think: Man, you look like complete crap!».

The (over-)weight was also a problem physically. Many reported limited flexibility, having become cumbersome, or even experiencing pain. Considering the emotional and physical burdens, the participants struggled with their weight and engaged in losing weight. However, the energy and motivation with which they approached the topic varied greatly and showed itself in two groups.

#### Losing Weight Requires the Right Headspace

Several participants had given some thought to what they could do to lose weight: Pay attention during meals, reduce portion size or carbohydrates, buy fitness equipment or join a gym. Although these ideas were clearly stated, the narratives remained vague: «I’m trying now to get back to that weight. With food, maybe eat a little less […] and maybe being more active.» (Katja).

Some tried to transform an idea into action but were often frustrated in daily life. Either the individual was unable to resist the temptation of delicious foods or the need for quiet and recuperation was greater than the urge to be active. Added to this was a certain lethargy due to various physical conditions such as pain or lack of stamina. Because the plans to lose weight were undertaken so half-heartedly, if at all, they met with little or no success. Many realized in retrospect that their plans to lose weight were not progressing because losing weight requires the right headspace for which you have to «overcome your “inner laziness”» (Margret). For some, their ambition was constrained by, as they explained, them having struggled with their weight even pre-LTx. Ultimately, those affected concluded for themselves that, given all that they had lived through, their present situation was acceptable. The awareness of being alive lessened the motivation to invest more energy to lose the bothersome extra weight.

#### All Attempts to Lose Weight Fail

Some participants attempted to lose weight by means of strict dieting and self-discipline. While everyone in this group lost a few kilos, they were either unable to reach their self-identified goal weight or, if they did, it was only a short time before they experienced a yo-yo effect, something they were unable to account for. In their own perception, they had tried to lose weight as best as they knew how and with deep commitment. The inexplicability of this failure and the realization that all of their efforts were in vain led to frustration and anger. They felt that they were at the mercy of a situation they were powerless to affect. Many felt that they had been abandoned in this situation—including by the LTx center’s healthcare professionals. Participants regarded the discussions at their follow-up appointments at the hospital as confusing and deemed the nutritional recommendations unhelpful: «You can forget it, I already know all of that and have done it for years» (Lara).

The original intention to lose weight began to fall by the wayside as failure and disappointment took a toll. The participants chose to confront the situation in various ways. Some adapted their goal to the new circumstances: at least do not gain more weight. Others, like Marlene, questioned the basic concept of weight loss: «What is the good of all of this to me?».

Memories of their illness, the transplantation, their recovery and the awareness of their own survival have been burned into their memory. The issue of excess weight lost its magnitude in comparison to what they had experienced. Rather, it became something they were able to come to terms with, borne out in the statements of their family members and social contacts. Physical and emotional burdens were brushed aside and a space was created for an awareness of having gotten through a difficult time. This insight not only offered consolation for the failure to lose weight; it also curbed the impulse to engage further with the topic. Having survived the severe illness served as a welcome justification and enabled a more forgiving relationship to their own overweight body: «You have to make the best of these sorts of things. But I always say to myself: It could be worse, at least I’m still around.» (Marlene).

## Discussion

Our findings highlight how patients put into perspective their lived experience of being an LTx survivor with the perception of post-LTx weight gain. *The Main Thing is to be Alive* Section was captured as a recurrent yet multifaceted conclusion in the three other themes, thereby shaping the patients’ perceptions of weight gain and coping mechanisms. Professionals should be aware of the dynamics to support patients in weight management. Based on the participants’ perceptions about a lack of support from healthcare professionals, we also provide clinical implications and suggestions for education and self-management support, based on the TTM in Table 3 (16, 17).

**TABLE 3:** Education and self-management support by healthcare professionals based on the Transtheoretical Model (TTM). The recommendations are based on the authors’ clinical experiences and the TTM, which describes stages of change: precontemplation, contemplation, preparation, action, maintenance and termination (16, 17). The stages represent a time dimension, although people may advance through the stages non-linearly. Progressing through the stages is accompanied by (overt or covert) activities that are described as processes of change (e.g., consciousness raising, self-reevaluation, environmental evaluation, stimulus control). Based on these core constructs, each stage requires a distinct intervention approach.

Theme	Transtheoretical model			Education and self-management support
	Stage of change	Process of change	Aim	
The arduous path back to living	Not applicable	Not applicable		• Priority is the physical and emotional recovery after LTx. Management of unplanned weight gain is most probably less important
				• Focus on relationship building during the frequent follow up appointments in the LTx center
A pleasurable new phase of life	Precontemplation: A person does not intend to take any action to prevent weight gain in the near future (usually described as 6 months)	Consciousness raising	Increase awareness on causes, consequences and potential treatment	Provide information on
				• Short- and long-term evolution of weight after LTx
				• Factors associated with weight gain in general
				• Body composition: offer repeated measurements to assess and specify the evolution of weight gain (e.g., increasing muscle mass or fat)
				• Risk of developing new-onset obesity and its associated outcomes after LTx (e.g., cardiovascular and metabolic comorbidities)
				• Concept of energy balance (calory consumption and expenditure)
				• Physical activity and healthy eating
				• Importance of self-monitoring of weight
				• The advantage of preventing excessive weight gain instead of losing weight afterwards
				Provide feedback
				• It may be important to acknowledge the patient’s healthy appearance with the regained weight. However, healthcare professionals should also critically question this development
				• Focus the communication on empowerment and self-management to intensify relationship building
I am allowed to look like this now	Contemplation: A person intends to take action within the next 6 months	Self-reevaluation	Facilitate the person’s assessment that behavior change is part of the own identity	• Assess the perception of weight gain and a potential burden during clinical follow-ups
				• Be aware of and listen to patient’s talking about pro and con arguments for changing their behavior
				• Identify the motivation, barriers and facilitators for behavior change
	Preparation: A person intends to take action within the next 30 days or has taken some behavioral steps already			• Define individual goals regarding the patients’ behavior (e.g., eating or activity) or weight loss (e.g., target weight)
				• Make sure that goals are specific, measurable, achievable, relevant, and time bound. Pay special attention to feasible goals regarding activity in case of functional impairment
				• Identify strategies to achieve the goals
				• Plan timely follow-up appointments
				• Evaluate the involvement of a nutritionist and physiotherapist
	Action stage: A person has changed the behavior for less than 6 months	Self-liberation	Support the persons commitment to change	• Provide feedback on achievement and celebrate the success
				• Strengthen the patient’s self-efficacy and self-consciousness

*The Arduous Path Back to Living* Section characterized the emotional course of the main theme. Our participants’ illness and recovery trajectory matched with previous descriptions. Life pre-LTx was dominated by distressing complications associated with a decreased quality of life and frequent hospitalizations, turning it into an unpredictable roller-coaster (24-26). Post-Tx, patients experienced increased physical functioning, emotional health and quality of life, contributing to the perception that the LTx was a salvation, miracle or gift (27-29). Those strong analogies emphasize the meaning of LTx for those affected. This meaning was also highlighted in our study, and the intense experiences framed the patients’ subsequent perception of their weight.

*A Pleasurable New Phase of Life* Section characterized the joyful part of the main theme, which was accompanied by weight gain. Participants in previous studies named increased food intake and improved appetite as main reasons for weight gain (9, 10). This finding contrasts to those in our narratives, where almost everyone insisted that their eating habits had returned to those pre-LTx. Nonetheless, this effect may have contributed to weight gain due to the concurrent decrease in physical activity, resulting in an energy imbalance between calory consumption and expenditure (30). Reduced activity is indeed common after LTx. Recipients have worse physical functioning compared to the general population (27), and only 45% meet the recommended physical activity levels (31). Although our participants were well aware of their inactivity, they did not prominently mention the idea of adapting their food intake accordingly. Moreover, as they did not regularly weigh themselves, the increased weight came as a surprise to most of them. This behavior should be targeted in interventions because self-monitoring of weight is crucial in successful weight management (32).

Another remarkable aspect was the participants’ appreciation of weight gain. As a body composition measurement was not available, the participants’ weight gain could not be specified in muscle mass, which would be positive and desired, or increasing fat mass, which would be associated with negative health outcomes such as cardiovascular or metabolic comorbidities (5-8). But in general, weight gain has been associated with decreased physical health-related quality of life (33), while obesity has been consistently associated with depression (34). None of our participants mentioned overweight or obesity in connection with potential negative health outcomes. In contrast, even if weight gain had led to overweight or obesity, the gain was equalized with looking healthy. Health seemed to be defined as the absence of liver disease, which remains an assumption as we did not further explore this topic. Nonetheless, weight gain was a visible sign of having survived the severe illness. Increased energy and wellbeing were indicators of recovery and normality. The importance of those reference points should not be underestimated. Patients in another study described the return to a self-defined normality as a milestone after LTx (28). Patients’ appreciation of the increased body weight combined with, 1) having no coherent explanation for weight gain, 2) the lack of awareness about intervening appropriately, and 3) not linking the excessive weight to potential negative outcomes present an opportunity for early and preventive interventions (Table 3).

*I am Allowed to Look Like This Now* Section characterized the consoling part of the main theme, which was visible in both groups, who differed with regard to the motivation to tackle weight gain. The vague wording by participants in the “Losing weight requires the right headspace” group indicated ambivalence. Although they felt physically and emotionally uncomfortable, they were not sufficiently triggered to engage in effective weight loss behaviors (e.g., reduce calorie intake, increase activity). Participants in the “All attempts to lose weight fail” group tried hard to lose weight or maintain weight loss, unfortunately unsuccessfully. Although our participants felt frustrated, angry and deserted by the LTx team, no-one mentioned in the interview to have actively sought additional support. However, weight loss and its maintenance are hard to achieve due to physiological compensation mechanisms (35), even with professional support. A telehealth-delivered lifestyle program combined Mediterranean diet with aerobic and resistance exercise after LTx (36). Although the intervention group lost weight over 12 weeks and the controls did not (mean −1.8 kg vs. +0.1 kg), the results show that weight loss comes in small steps and takes time. Although guidelines consistently advise to prevent weight gain instead of trying to lose weight afterwards (37), studies examining preventive weight interventions after LTx are lacking. Participants who contributed experiences to this theme expressed stress and burden due to the increased weight and unsuccessful weight loss attempts. Yet our analysis left us wondering if the participants’ negative narratives were really part of their daily life or if they were rather nudged by our interview. Our reservations arose because it seemed as if participants in both groups did not feel enough pressure to more rigorously lose weight by finding alternatives to the previous failed attempts. Instead, the theme *I am allowed to look like this now* emerged as an insight. This may have comforted participants but it bares the potential of a killer argument because it may stop further engagement in weight loss. Professionals should proactively assess the TTM’s stages of change to provide targeted support (Table 3).

Our study findings should be interpreted in light of some limitations. In qualitative studies results are not generalizable to all patients. The categorization of the weight category at time of the interview relied on a self-reported weight measure and was not verified by an objective measurement performed by a healthcare professional. The weight category at LTx was not considered as inclusion criteria, which contributed to the heterogeneity of the group. The analysis did not consider disease etiologies to account for various perspectives on weight gain. Future studies in distinct subgroups are needed to explore potential differences in experiences and coping strategies. As we only included German-speaking patients, we lack understanding of how people with other ethnic or cultural backgrounds experience post-LTx weight gain. They may have different perceptions resulting in a need for other supportive interventions diverse.

## Conclusion

Exploring patients’ experiences with weight gain after LTx revealed the importance of having survived the severe illness, which shaped perceptions of and coping with weight gain. After suffering during the course of LTx, the weight increase was initially appreciated and equated with health. For some participants, ongoing weight gain led to an emotional and physical burden, which was brushed away by the comforting insight *I am allowed to look like this now.* As this argument might hinder further engagement in weight loss interventions, professionals should be aware of the need for early interventions that address patients’ specific needs related to weight gain after LTx.

## Data Availability

The raw data supporting the conclusion of this article will be made available by the authors, without undue reservation.

## Abbreviations

BMI, body mass index; COREQ, Consolidated Criteria for Reporting Qualitative Health Research; LTx, liver transplantation; TTM, transtheoretical model.
