# Supplementary material for: The Main Thing is to be Alive—Exploring Patients’ Experiences With Weight Gain After Liver Transplantation: A Qualitative Study
Source: Transpl Int. 2022 Apr 14;35:10256. doi: 10.3389/ti.2022.10256 (PMC9046544; doi:10.3389/ti.2022.10256)
Supplement: Supplementary file 1 [file DataSheet2.docx]

**Interview guide**

**Introduction to the topic:**

State the aim of the study: "It is well known that patients gain weight after liver transplantation. With this study, we want to investigate your experience with weight gain."

**Introductory question:**

"People affected by liver transplantation report that their lives have changed. Can you please tell me how your daily life and routine have changed since your liver transplant?"

| **Possible guiding questions** | **Weight** | - You have gained weight after liver transplantation. How does gaining weight affect your daily life? - What do you think is the reason you gained weight after your liver transplant? - What reasons were there that made it harder for you to keep the weight off (lose weight)? Was there anything that made it easier? - What skills would you have needed to keep you from gaining weight (keeping weight off)? - How did those around you react to your weight gain? |
| --- | --- | --- |
|  | **Physical activity** | - What impact did physical activity have on your weight gain? - Please describe for me the activities you did before the liver transplant? What were the activities after liver transplant? - What motivated you to be more physically active? What incentives were there to be physically active? - What situations were there when you could not be physically active? Can you tell me what made it difficult? |
|  | **Nutrition** | - Please describe for me how your diet has changed since your liver transplant? (What do you like to eat? Who does the grocery shopping? Who does the cooking in your home? How has your appetite changed?) - Can you describe to me your routine/situation at home for lunch/dinner? - What impact does your diet have on weight gain? - Can you describe to me situations when it is difficult (easy) to eat healthy? - What incentives were there to (not) eat healthy? |
| **Possible in-depth questions** |  | - Can you describe this statement to me in a little more detail? - Can you give me a concrete example? - What was that like for you? - What do you mean by that? - That's interesting, can you be more specific? |
